# Comparison of Serum MicroRNA21 and Tumor Markers in Diagnosis of Early Non-Small Cell Lung Cancer

**DOI:** 10.1155/2016/3823121

**Published:** 2016-01-11

**Authors:** Mingzhong Sun, Jiangxiang Song, Zhongwei Zhou, Rong Zhu, Hao Jin, Yuqiao Ji, Qiang Lu, Huixiang Ju

**Affiliations:** Department of Clinical Laboratory, Affiliated Yancheng Hospital, School of Medicine, Southeast University, Yancheng, Jiangsu 224001, China

## Abstract

*Objective.* To compare the clinical value of serum microRNA21 (miR21) and other tumor markers in early diagnosis of non-small cell lung cancer (NSCLC).* Methods.* Serums carcinoembryonic antigen (CEA), cytokeratin 19 fragment (CYFRA21-1), neuron-specific enolase (NSE), and miR21 were detected in 50 NSCLC cases and 60 healthy control individuals.* Results.* Average serums miR21, CEA, NSE, and CYFRA21-1 levels were significantly higher in the case group than in control group (*P* < 0.01). Analysis of areas under the receiver operating characteristic (ROC) curve (AUC) revealed that CEA had the highest diagnostic efficiency for NSCLC. Serums miR21 and CYFRA21-1 levels were significantly lower at TNM stages I-II than stages III-IV (*P* < 0.05). Further, logistic multivariate regression analysis showed that the incidence of early NSCLC (TNM stages I-II) was correlated with serums CYFRA21-1 (OR = 1.076) and miR21 (OR = 2.473) levels (*P* < 0.05). By AUC analysis, miR21 had the highest diagnostic efficiency for early NSCLC, and single or combined detection of serums CYFRA21-1 and miR21 levels showed improved diagnostic efficiency for joint detection of both markers.* Conclusions.* Serum miR21 could serve as an important marker for auxiliary diagnosis of early NSCLC, while joint detection of serums miR21 and CYFRA21-1 levels could improve diagnostic efficiency.

## 1. Introduction

The annual morbidity rate of non-small cell lung cancer (NSCLC) has been increasing in recent years. Both in China and worldwide, NSCLC has become one of the most lethal tumor types [1]. With clinical application of newer molecular targeted drugs, such as gefinitib, erlotinib, and crizotinib, platinum-containing two-medicine combination and targeted therapy regimens have somewhat improved the therapeutic outcome of late-stage NSCLC [2–4]. However, the survival rate and overall prognosis of patients with late-stage NSCLC remain relatively poor [5]. Therefore, improving early diagnosis is key to advancing the prognosis of NSCLC patients.

Biopsy by bronchoscope, mediastinoscope, or thoracentesis is the most reliable method to diagnose NSCLC. However, these techniques have many contraindications in application and thus are not practical for early screening and continuous monitoring of the disease. Serum marker detection—with advantages including easy operation, low price, noninvasiveness, accessibility of samples, and ability for continuous monitoring—is a high-profile topic for auxiliary diagnosis of early NSCLC [6]. Clinical studies have examined various indicators, such as carcinoembryonic antigen (CEA), cytokeratin 19 fragment (CYFRA21-1), neuron-specific enolase (NSE), carbohydrate antigen (CA-199), cytokeratin 5/6 (CK 5/6), cytokeratin HMW (CK-HMW), thyroid transcription factor-1 (TTF-1), and cytokeratin 8/18 (CK 8/18). However, no reliable and independent indicator for early diagnosis of NSCLC has been found [7], so joint marker detection is the main measure to improve diagnosis of early NSCLC using serum markers.

During the initiation and development of NSCLC, driver genes that induce and maintain molecular changes of malignant tumors, such as epidermal growth factor receptor (EGFR), anaplastic lymphoma kinase (ALK), fibroblast growth factor receptor 1 (FGFR1), and phosphoinositide 3-kinase catalytic subunit A (PIK3CA), play an important role [8]. Previous studies verified that, during gene expression and evolution, highly conserved and stable microRNAs (miRs) help regulate expression of carcinogenic genes and are closely associated with cell proliferation and differentiation as well as the occurrence, development, invasion, and metastasis of malignant tumors [9, 10].

Recent studies have indicated that miRs participate in the occurrence, development, and prognosis of pulmonary cancer and have similar effects as protooncogenes or tumor-suppressing genes. In pulmonary cancer tissues, miRs have unique expression profiles and participate in multiple processes, such as regulating tumor angiogenesis [11, 12]. Therefore, miRs may be useful biological markers for early diagnosis, targeted therapy, and evaluation of clinical prognosis of NSCLC. In particular, previous studies have shown that miR21 expression is deregulated in many cancers including NSCLC, in which its expression is associated with poor patient outcome [13–15]. miR21 appears to exert prooncogenic effects by targeting various genes within each of the different hallmarks of cancer (for review, see [Buscaglia and Li]) [16]. In particular, upregulation of miR21 appears to suppress apoptosis by targeting various players in apoptosis pathways, such as by downregulating the tumor suppressor PTEN [16, 17]. Its potential to promote NSCLC makes miR21 a potential novel biomarker for this cancer. Therefore, this study comparatively analyzed the value of miR21 compared to tumor markers CEA, NSE, and CYFRA21-1 for early diagnosis of NSCLC.

## 2. Subjects and Method

### 2.1. Study Subjects

The study included a case group of 50 NSCLC patients admitted to Affiliated Yancheng Hospital, School of Medicine, Southeast University (Yancheng, China) from January 2013 to January 2014. Patients underwent pulmonary tumor resection, and NSCLC was confirmed by postoperative histopathology. Patients did not receive radiotherapy or chemotherapy before surgery. The case group included 38 men and 12 women, 45–81 years old with a mean age of 66.9 ± 8.7 years. Of the 50 cases, 29 were squamous cell carcinomas and 21 were adenocarcinomas. Analysis of TNM staging indicated that 7 tumors were stage I, 15 tumors were stage II, 19 tumors were stage III, and 9 tumors were stage IV.

The study also included a control group of 60 individuals verified to be healthy volunteers by physical examination in Affiliated Yancheng Hospital, School of Medicine, Southeast University (Yancheng, China) during the same period. The control group included 47 men and 13 women, 42–78 years old with a mean age of 62.4 ± 7.9 years. Age and gender significantly differed between the two groups (*P* > 0.05). All subjects included in the study provided written informed consent. The study protocol was approved by the Medical Ethics Committee of Affiliated Yancheng Hospital, School of Medicine, Southeast University (Yancheng, China).

### 2.2. Serum Marker Assessment

Fasting peripheral vein blood specimens were collected from subjects. For the case group, specimens were collected on the day after subjects were admitted to the study. For the control group, specimens were collected on the same day as the physical examination. Blood samples were centrifuged to collect serum samples. Levels of CEA, NSE, and CYFRA21-1 were detected in serum specimens with a double-antibody sandwich magnetic particle chemiluminescent method using matched kits with the Roche (e601, Basel, Switzerland) automatic electrochemical luminescent immunoassay analyzer per manufacturer's instructions.

Real-time fluorescent quantitative PCR (RT-PCR) was used to detect and compare expression of miR21 in serum specimens. RNA was extracted with an RNA extraction kit (Applied Biosystems, Foster City, California, USA) at short notice, and RNA was immediately stored at −80°C. To detect miR expression, samples were reverse transcribed using a reverse transcription kit (ABI, USA). Reactions were performed in 15 *μ*L total volumes, containing 0.15 *μ*L of 100 mmol/L dNTPs; 1 *μ*L of MultiScribe reverse transcriptase; 1.5 *μ*L of 10X reverse transcription buffer; 0.19 *μ*L of RNase inhibitor; 4.16 *μ*L of sterile water; 5 *μ*L of total RNA diluted 1 : 1 with sterile water; and 3 *μ*L of primer (20 *μ*M). The primer sequence, obtained from the Sanger miR sequence database, was 5′-GTGCAGGGTCCGAGGT-3′. Samples were reverse transcribed at 16°C for 30 min, 42°C for 30 min, and 85°C for 5 min and held at 4°C in the PCR Thermal Cycler Dice (Code TP600, TaKaRa).

Quantitative detection was performed on reverse-transcribed products in 20 *μ*L reactions, containing 10 *μ*L of TaqMan Universal Master Mix; 4 *μ*L of sterile water; 1 *μ*L of TaqMan MicroRNA Assay reagent; and 5 *μ*L of reverse transcription product diluted 1 : 1 with sterile water. Reactions were amplified on a Roche Cobas z 480 RT-PCR system, using the following conditions: 95°C for 10 min and 40 cycles of 95°C for 15 s and 60°C for 1 min. PCR system software was used to produce baseline and threshold values, with miR16 as the internal reference [18–21]. Cycle threshold (Ct) values correspond to the number of cycles required for the amplified product to reach a critical threshold of detection. ΔCt refers to the difference between miR21 and miR16 Ct values. 2^−ΔΔCt^ refers to the expression level of miR21.

### 2.3. Statistical Analysis

The SPSS 13.0 statistical package (IBM Corp., Armonk, NY, USA) was utilized to establish a database of all study data. Measurement data were expressed as mean ± standard deviation. Independent sample *t*-test was used for comparisons between case and control groups. Enumeration data were expressed as percentages. Area under receiver operating characteristic (ROC) curve (AUC) was used to compare serum marker diagnostic efficiency. *P* < 0.05 was considered statistically significant.

## 3. Results

### 3.1. Comparison of Serum miR21, CEA, NSE, and CYFRA21-1 Levels of Study Participants

Serum miR21, CEA, NSE, and CYFRA21-1 levels were, on average, significantly higher in patients with NSCLC than in control individuals (Table 1).

### 3.2. Correlation between Clinical Features and Serum miR21, CEA, NSE, and CYFRA21-1 Levels of NSCLC Patients

Serum miR21, CEA, NSE, and CYFRA21-1 levels did not significantly differ with gender or age of NSCLC patients or with tumor pathology (Table 2). In addition, serum CEA and NSE levels of NSCLC patients did not significantly differ with clinical stage of disease. However, serum miR21 and CYFRA21-1 levels of NSCLC patients at TNM stages I-II were significantly lower than those of patients at TNM stages III-IV (*t* = 2.575, −2.301, resp.; *P* < 0.05).

### 3.3. Diagnostic Value of Serum miR21, CEA, NSE, and CYFRA21-1 Levels for NSCLC

**Figure 1:**
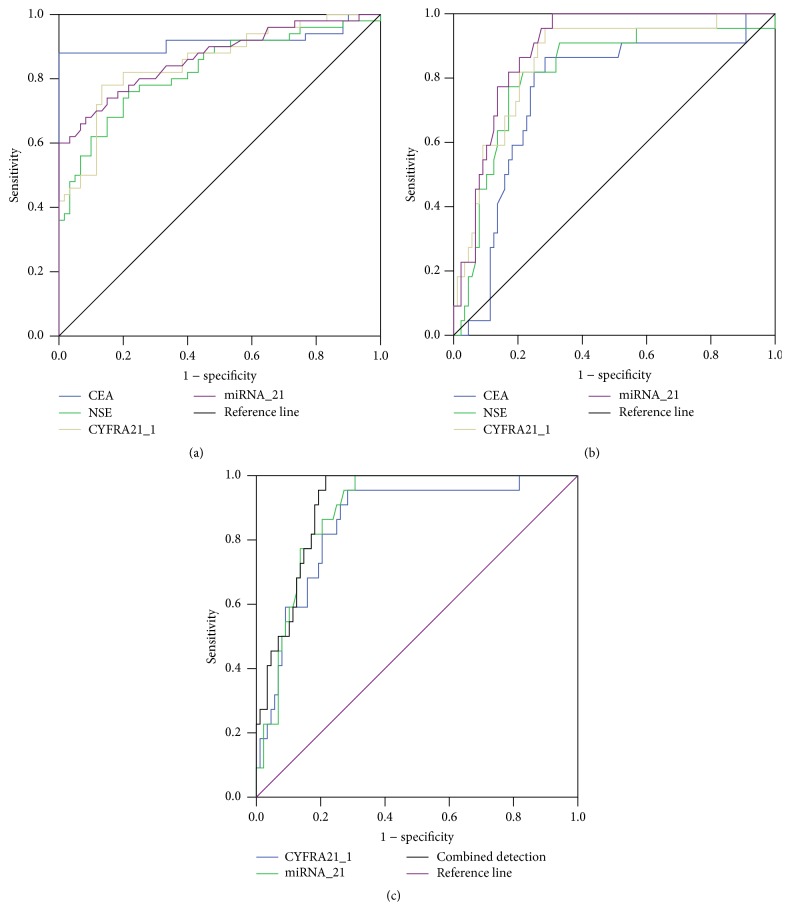
Receiver operating characteristic (ROC) curve of using serum miR21, CEA, NSE, and CYFRA21-1 levels. (a) ROC curve of using serum miR21, CEA, NSE, and CYFRA21-1 levels to diagnose NSCLC; (b) ROC curve of using serum miR21, CEA, NSE, and CYFRA21-1 levels to diagnose early NSCLC; (c) ROC curve for single detection of serum miR21 or CYFRA21-1 level and joint detection of both markers to diagnose early NSCLC.

AUC of using serum miR21, CEA, NSE, and CYFRA21-1 levels to diagnose NSCLC was 0.918, 0.826, 0.853, and 0.866, respectively. CEA had the highest diagnostic efficiency for NSCLC (Figure 1(a), Table 3).

### 3.4. Diagnostic Value of Serum miR21, CEA, NSE, and CYFRA21-1 Levels for Early NSCLC

After removing the data for TNM stages III-IV, the AUC of using serum miR21, CEA, NSE, and CYFRA21-1 levels to diagnose early NSCLC (TNM stages I-II) was 0.752, 0.806, 0.843, and 0.882, respectively. miR21 had the highest diagnostic efficiency for early NSCLC (Figure 1(b), Table 4). Logistic multiple regression analysis was utilized to analyze relevancy between the four serum markers and early NSCLC. The results indicated that occurrence of early NSCLC was significantly correlated with serum CYFRA21-1 (OR = 1.076; Wald *χ* ^2^ = 4.025) and miR21 (OR = 2.473; Wald *χ* ^2^ = 9.153) levels (*P* < 0.05) (Table 5).

Comparison of joint detection of serum CYFRA21-1 and miR21 levels with single detection of either marker indicated that AUC of single detection for CYFRA21-1, single detection for miR21, and joint detection of both markers were 0.843, 0.872, and 0.909, respectively (Figure 1(c), Table 6).

## 4. Discussion

This study indicates that, on average, serum miR21 and CYFRA21-1 levels were lower in patients with NSCLC at TNM stages I-II than NSCLC at TNM stages III-IV. However, logistic multiple regression analysis indicated that early NSCLC (TNM stages I-II) was associated with serum CYFRA21-1 and miR21 levels. AUC analysis showed that miR21 had the highest diagnostic efficiency for early NSCLC. Analysis of the utility of single or joint detection of CYFRA21-2 and miR21 indicated that miR21 and CYFRA21-1 expression were somewhat related and played an auxiliary role in diagnosis of early NSCLC. In single marker detection, the efficiency of serum miR21 levels to diagnose early NSCLC was higher than other examined markers. Therefore, miR21 could serve as an important serum marker for auxiliary diagnosis of early NSCLC, while joint detection of serum miR21 and CYFRA21-1 levels could improve diagnostic efficiency.

CEA is a polysaccharide protein complex antigen produced during embryonic development. CEA is a broad-spectrum tumor marker because it is expressed in multiple tumors and is closely associated with malignant tumors. The marker is mainly used in clinical practice to assist diagnosis, assess tumor staging and lesion degree, monitor treatment, and predict recurrence of malignant tumors. Recent studies have verified that serum CEA expression in NSCLC patients is related to* EGFR* mutation rate and can serve as an indicator to guide targeted therapy [22]. Serum CEA level also is an independent factor that informs prognosis of NSCLC patients. Increased CEA expression in NSCLC patients is related to decreased postoperative survival rate, and serum CEA levels can help predict prognosis of NSCLC patients after surgical tumor resection. For patients with recurrence of advanced NSCLC with inaccessible tissues, CEA expression can be used as a referential indicator to predict therapeutic efficacy of EGFR tyrosine kinase inhibitors [23]. However, we did not observe significantly different serum CEA levels in patients at different TNM stages in this study.

Considering a main driving force of miR transcription, miR21 plays an important regulatory role in cell proliferation, differentiation, and apoptosis and has a close relationship with tumor biological behaviors, such as growth, invasion, and migration [24]. Previous studies have verified that miR21 expression in pulmonary carcinoma tissues is significantly higher than in tissues from healthy individuals [15, 18]. In addition, recurrence and metastasis of pulmonary carcinomas with high miR21 expression are significantly higher than those with low miR21 expression, and thus high-miR21-expressing pulmonary carcinomas result in decreased patient survival [25]. When compared with relevant studies, the miR21 results of this study indicate a difference in terms of sensitivity estimation. A meta-analysis of 8 studies, including 600 patients and 440 study control subjects [26], previously measured the concomitant sensitivity of using serum miR21 to detect and diagnose early pulmonary carcinoma at 72%, concomitant specificity at 84%, and AUC at 0.898. Differing results obtained in the current study can be attributed to its smaller sample size from the meta-analysis and selectivity for only NSCLC rather than other pulmonary carcinomas. Our findings are consistent with other findings in which miR21 expression could identify individuals with lung cancer [27, 28]. Another meta-analysis determined the value of measuring miR21 expression in serum for a variety of cancer types. The authors concluded that serum miR21 measurement appears to be a valid predictor for the diagnosis of early-stage cancers and has higher diagnostic accuracy in Asians than in Caucasians [29]. Thus, miR21 may be an important translational tool for clinical diagnosis and prognosis.

The results of this study also indicate that CYFRA21-1 is valuable for diagnosis of early NSCLC. CYFRA21-1 is an epithelial tumor marker with abnormal expression associated with occurrence and development of multiple common malignant epithelial carcinomas, such as pulmonary carcinoma, nasopharyngeal carcinoma, esophageal carcinoma, laryngeal carcinoma, urinary bladder carcinoma, mammary adenocarcinoma, oophoroma, and colorectal cancer [30]. Studies have verified that serum CYFRA21-1 levels are related to indicators such as imageological disease control (DC) and progression-free survival (PFS) of pulmonary carcinoma patients [31]. CYFRA21-1 also can sensitively reflect changes in imageological tumor volume and size. Among patients with progressive NSCLC with elevated CYFRA21-1 levels, changes in CYFRA21-1 expression before and after one-cycle chemotherapy can be used to predict imageological DC and PFS and can provide early evidence for adjusting the therapeutic regimen. CYFRA21-1 expression also can serve as an alternative index to assess chemotherapeutic efficacy of patients with progressive NSCLC [32].

In this study, NSE showed no diagnostic value for NSCLC, especially early NSCLC. NSE is a marker present in neurons and neurosecretory cells and can sensitively reflect central nervous system injuries, such as acute cerebrovascular disease, epilepsy, acute brain trauma, and neonate hypoxic-ischemic encephalopathy. As a tumor marker, NSE is mainly associated with specificity of neuroblastoma and small cell lung cancer (SCLC), and its ability to diagnose NSCLC is inferior to other tumor markers [33]. NSE therefore is often clinically used together with another marker, such as CEA, CYFRA21-1, CA199, CA125, or SCC, in joint detection for differential diagnosis, chemotherapeutic efficacy evaluation, and prognostic evaluation of patients with pulmonary carcinomas [34].

A limitation of this study is its small sample size. Larger populations should be investigated to confirm these findings and further establish the utility of miR21 expression as a clinical tool. Work that demonstrates the action of miR21 on targets involved in apoptosis (reviewed by [16]) suggests that our findings for increased miR21 expression in NSCLC likely implicate it in suppressing apoptosis. Indeed, Ono et al. showed that suppression of miR21 in human cell lines induces apoptosis [28], providing an important molecular foundation for further investigation of miR21 as a clinical target.

## 5. Conclusion 

Among abnormally expressed tumor markers in NSCLC patients, miR21 has high efficiency in diagnosing early NSCLC and therefore can serve as an important serum marker in auxiliary diagnosis. Joint detection of miR21 with serum CYFRA21-1 can further improve diagnostic efficiency for early NSCLC.

## Figures and Tables

**Table 1:** Serum miR21, CEA, NSE, and CYFRA21-1 levels of study participants.

Group	*n*	CEA (ng/mL)	NSE (ng/mL)	CYFRA21-1 (ng/mL)	miR21 (arbitrary units)
Case	50	35.94 ± 19.63	23.66 ± 12.79	12.92 ± 9.15	2.14 ± 1.14
Control	60	4.47 ± 2.52	10.01 ± 6.14	3.24 ± 3.43	0.77 ± 0.45

*t*		11.257	6.914	7.073	8.014
*P*		<0.01	<0.01	<0.01	<0.01

^*∗*^miR21 expression was normalized against miR16 expression and is described in relative units.

**Table 2:** Correlation between clinical features and serum miR21, CEA, NSE, and CYFRA21-1 levels of NSCLC patients.

Clinical feature	Variable	*n*	CEA (ng/mL)	NSE (ng/mL)	CYFRA21-1 (ng/mL)	miR21 (arbitrary units)
Gender	Male	38	33.13 ± 20.25	22.38 ± 13.45	12.65 ± 9.84	2.04 ± 1.19
	Female	12	44.88 ± 14.96	27.71 ± 9.80	13.78 ± 6.79	2.49 ± 0.94

	*t*		−1.851	−1.266	−0.372	−1.189
	*P*		0.070	0.212	0.712	0.240

Age	>60	21	38.22 ± 19.80	25.90 ± 13.62	15.70 ± 9.58	2.23 ± 1.14
	≤60	29	34.30 ± 19.70	22.03 ± 12.13	10.90 ± 8.42	2.08 ± 1.16

	*t*		0.692	1.059	1.878	0.461
	*P*		0.492	0.295	0.066	0.647

Pathology	Squamous cell carcinoma	29	40.47 ± 20.44	22.41 ± 11.62	12.82 ± 8.90	2.33 ± 1.14
	Adenocarcinoma	21	29.70 ± 16.99	25.38 ± 14.36	13.06 ± 9.70	1.89 ± 1.12

	*t*		1.970	−0.806	−0.092	1.360
	*P*		0.055	0.424	0.927	0.180

TNM stage	I-II	22	33.16 ± 17.68	21.50 ± 13.76	10.39 ± 8.99	1.80 ± 1.18
	III-IV	28	38.14 ± 21.10	26.41 ± 11.14	16.14 ± 8.50	2.59 ± 0.95

	*t*		−0.888	1.360	2.301	2.575
	*P*		0.379	0.180	0.026	0.013

^*∗*^miR21 expression was normalized against miR16 expression and is described in relative units.

**Table 3:** Diagnostic value of serum miR21, CEA, NSE, and CYFRA21-1 levels for NSCLC.

Test result variable	Area	Standard error	Asymptotic significance	Asymptotic 95% confidence intervals	
				Lower bound	Upper bound
CEA	0.918	0.034	0.000	0.851	0.985
NSE	0.826	0.041	0.000	0.747	0.906
CYFRA21-1	0.853	0.037	0.000	0.780	0.925
miR21	0.866	0.036	0.000	0.796	0.936

**Table 4:** Diagnostic value of serum miR21, CEA, NSE, and CYFRA21-1 levels for early NSCLC.

Test result variable	Area	Standard error	Asymptotic significance	Asymptotic 95% confidence interval	
				Lower bound	Upper bound
CEA	0.752	0.058	0.000	0.638	0.866
NSE	0.806	0.051	0.000	0.723	0.914
CYFRA21-1	0.843	0.041	0.000	0.768	0.938
miR21	0.882	0.028	0.000	0.821	0.940

**Table 5:** Multiple-factor analysis of relevancy between serum miR21, CEA, NSE, and CYFRA21-1 levels and early NSCLC.

Variable	*B*	Standard error	Wald	df	*P*	Exp(*B*)
CEA	0.001	0.016	0.001	1	0.972	1.001
NSE	0.032	0.031	1.189	1	0.293	1.026
CYFRA21-1	0.079	0.041	4.025	1	0.046	1.076
miRNA-21	0.963	0.300	9.153	1	0.002	2.473
Constant	−4.615	0.793	24.075	1	0.000	0.010

**Table 6:** Diagnostic value of single detection of serum miR21 or CYFRA21-1 level and joint detection of both markers for early NSCLC.

Test result variable(s)	Area	Standard error	Asymptotic significance	Asymptotic 95% confidence interval	
				Lower bound	Upper bound
CYFRA21-1	0.843	0.041	0.000	0.780	0.922
miRNA-21	0.872	0.030	0.000	0.851	0.936
CYFRA21-1 + miRNA-21	0.909	0.028	0.000	0.872	0.951
